# Treatment of water extract of green tea during kale cultivation using a home vertical farming appliance conveyed catechins into kale and elevated glucosinolate contents

**DOI:** 10.1016/j.crfs.2024.100852

**Published:** 2024-09-14

**Authors:** Young-Woong Ju, Su-Hyeon Pyo, So-Won Park, Chae-Ryun Moon, Seul Lee, Mzia Benashvili, Jai-Eok Park, Chu Won Nho, Yang-Ju Son

**Affiliations:** a Department of Food and Nutrition, Chung-Ang University, Anseong, 17546, Republic of Korea; b Smart Farm Research Center, Gangneung Institute of Natural Products, Korea Institute of Science and Technology (KIST), Gangneung, 25451, Republic of Korea

**Keywords:** Kale, Green tea, Vertical farm, Catechin, Glucosinolate

## Abstract

The growing interest in healthy diets has driven the demand for food ingredients with enhanced health benefits. In this study, we aimed to explore a method to enhance the bioactivity of kale using a home vertical farming appliance. Specifically, we investigated the effects of treating kale with a green tea water extract (GTE; 0.1–0.5 g/L in nutrient solution) for two weeks before harvest during five weeks of kale cultivation. GTE treatment did not negatively affect the key quality attributes, such as yield, semblance, or sensory properties. However, it led to the accumulation of bioactive compounds, epicatechin (EC) and epigallocatechin gallate (EGCG), which are typically absent in kale. In the control group, no catechins were detected, whereas in the GTE-treated group, the concentration of EC and EGCG were as high as 252.11 and 173.26 μg/g, respectively. These findings indicate the successful incorporation of catechins, known for their unique health-promoting properties, into kale. Additionally, GTE treatment enhanced the biosynthesis of glucosinolates, which are key secondary metabolites of kale. The total glucosinolate content increased from 9.56 μmol/g in the control group to 16.81 μmol/g in the GTE-treated group (treated with 0.5 g/L GTE). These findings showed that GTE treatment not only enriched kale with catechins, the primary bioactive compounds in green tea but also increased the levels of glucosinolates. This study, conducted using a home vertical farming appliance, suggests that bioactivity-enhanced kale can be grown domestically, providing consumers with a nutrient-fortified food source.

## Highlights

• Green tea extract (GTE) is used in kale cultivation to enhance its bioactivity.
• A home vertical farm appliance was used to increase the validity of our results.
• GTE treatment conveyed epicatechin and epigallocatechin gallate to kales.
• GTE treatment elicited an increase in total glucosinolate content in kale.
• GTE treatment of kale can counteract the effects of catechins and glucosinolates.

## Graphical abstract

**Figure:**
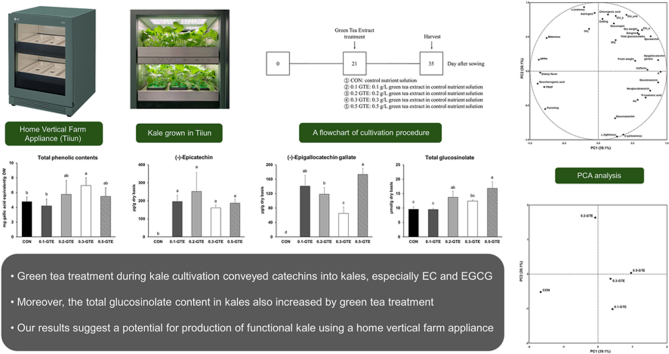

## 1 Introduction

Personalized nutrition has recently gained attention in dietary management, considering personal health status (Selvi et al., 2022). Personalized nutrition considers the biological characteristics of individuals, such as age, sex, genetic factors, and the presence of diseases (Hassoun et al., 2023; Kirk et al., 2021). Increases in the proportion of the elderly population and patients who struggle with chronic diseases have encouraged the progression of personalized nutrition (Hassoun et al., 2023); this highlights the requirement for appropriate dietary items that can improve individual health status. Plant-based foods are rich in nutrients and contain many bioactive components that regulate the metabolic pathways in the human body. Some functional herbs are used as food or dietary supplements and their applications support personalized nutrition and human wellness. Artificial cultivation has been performed to mass-produce these herbs. However, conventional farming has been encountering a scarcity of arable land areas because of soil erosion and pollution, and global climate change has compelled the diversion of long-standing farming systems (Khan, 2018). Moreover, conventional farming is highly influenced by uncontrolled environmental factors, which impede the production of high-quality, standardized harvests.

Vertical farming is an alternative cultivation system that grows crops indoors; therefore, it does not rely on sunlight or soil and is independent of external conditions (Jürkenbeck et al., 2019). Vertical farming commonly equips structures with multi-tiered shelves within the building, which helps increase the yield per unit area compared with the conventional method. The environmental conditions in vertical farming are artificially organized, and light, temperature, humidity, carbon dioxide concentration, and water are precisely controlled using information and communication technologies (Van Gerrewey et al., 2021). Precisely controlled cultivation conditions support the production of high-quality standardized crops. Therefore, vertical farming facilities have emerged in many countries, including Japan and Singapore (Benke and Tomkins, 2017). In addition, it enables novel innovations to fortify the nutrient content and functional activities of plants (Al-Kodmany, 2018; Jones Jr, 2016). The modulation of light sources is a popular approach, and the use of blue light in vertical farming has increased the amount of glucosinolates and phenolic compounds in leafy greens (Wong et al., 2020). Another classic way to increase the functional activity of plants is to treat them with certain minerals during cultivation. For example, Kim et al. (2018) reported that treatment of kale with NaCl and Na_2_SeO_3_ elevated isothiocyanate content and that harvested kale upregulated Nrf2 expression in HepG2 cell lines, which is a key modulator of the cellular antioxidative defense system. Our previous study also proposed an opportunity to produce crops for specific purposes using a vertical farming system, low-potassium kale, a suitable dietary ingredient for patients with renal dysfunction (Son et al., 2021).

Green tea is one of the most widely consumed beverages worldwide, and approximately 500,000 tons of green tea leaves are handled annually (Chacko et al., 2010). The leaves of *Camellia sinensis* are a source of green tea, undergo fixation and rolling to prevent enzymatic oxidation and increase the overall quality of green tea (Brimson et al., 2022; Satoh et al., 2005; Wang et al., 2018). Green tea is an essential source of catechins, which are polyphenolic compounds belonging to the flavan-3-ols (flavanols) family, and catechins contribute to the bitter and astringent tastes of green tea (Bernatoniene and Kopustinskiene, 2018; Komes et al., 2010). Catechins have high antioxidative activities, and numerous animal studies have revealed their anti-obesity and anti-diabetic effects (Chacko et al., 2010; Coimbra et al., 2006). Moreover, green tea also contains some purine alkaloids and plentiful phenolic compounds that enhance various bioactive properties, including anti-tumor activity and alleviation of inflammation and hypertension (Friedman et al., 2006; Imran et al., 2019; Singh et al., 2021).

*Brassica oleracea* var. *acephala* is a variety of kales and a popular leafy vegetable globally (Khalid et al., 2023). Kale has a higher proportion of unsaturated fatty acids than saturated fatty acids and possesses calcium with higher bioavailability than milk (Satheesh and Workneh Fanta, 2020). Kale possesses high fiber and vitamin C contents and is a good source of flavonoids, α-tocopherol, and β-carotene (Schmidt et al., 2010; Sikora and Bodziarczyk, 2012). Kale also has abundant riboflavin, folate, and vitamins K and A compared to other cruciferous vegetables; therefore, it is known to have various health benefits such as ophthalmologic problems, hepatic diseases, obesity, and diabetes (Šamec et al., 2019). Kale is a cruciferous vegetable containing glucosinolates, which are unique sulfur-containing active compounds (Casajús et al., 2021). Glucosinolates have various health benefits, including antioxidative stress, anti-inflammation, and anti-carcinogenic effects (Miękus et al., 2020). Because of the different bioactivities of green tea and kale, we anticipated that the functional activities would be highly increased if the unique bioactive components in green tea and kale could merge.

In the present study, we hypothesized that supplementation with green tea extract during kale cultivation would increase the functional activities of kale by absorbing active compounds from the green tea extract. If catechins can infiltrate kale, the bioactivity of catechins and glucosinolates can be combined in a single plant. Moreover, green tea extract could also act as an elicitor that stimulates and changes the expression of metabolic pathways in kale, especially for the synthesis of intrinsic secondary metabolites such as glucosinolates. As a cultivation system, we adopted a home vertical farming appliance to control environmental conditions precisely. We also anticipated that using home vertical farming appliances would enable people to obtain personally tailored harvests. Therefore, the characteristics (color, flavor, antioxidant capacity, and content of bioactive compounds) of green tea extract-treated kale were verified in this study to examine the feasibility of producing functional kale at home.

## 2 Materials and methods

### 2.1 Plant materials and growth conditions

**Fig. 1:**
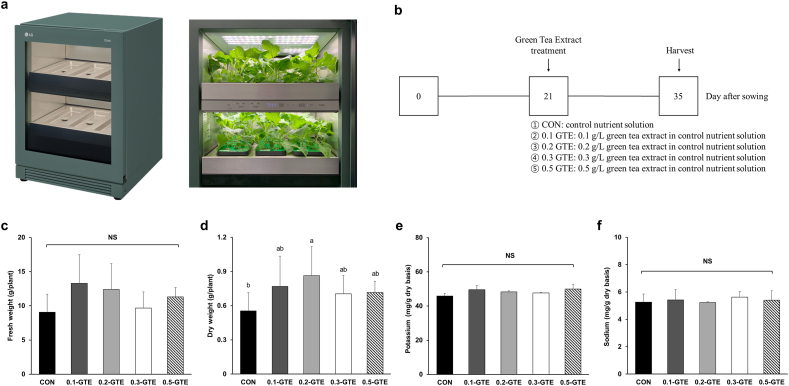
Pictures of a home vertical farming appliance (a), a flowchart of cultivation procedure (b), yield (fresh weight, c; dry weight, d), and mineral contents (sodium, e; potassium, f) of green tea-treated kales. Data represent the means ± SDs (n = 6, biologically independent plant sample groups). Different letters indicate significant differences within groups (p < 0.05). NS, not significant. Treatment with the basic nutrient solution was performed for the samples in the CON group; treatment with the nutrient solution containing 0.1, 0.2, 0.3, or 0.5 g/L of green tea water extract (GTE) was performed for the samples in the 0.1-GTE, 0.2-GTE, 0.3-GTE, and 0.5-GTE groups, respectively. (For interpretation of the references to color in this figure legend, the reader is referred to the Web version of this article.)

Commercial kale seeds (Manchoo collard, a cultivar of *Brassica oleracea* L. *var. acephala (DC.) Alef*) were obtained from Asia Seed Co. (Seoul, Korea). A home vertical farming appliance (Tiiun; LG Electronics, Seoul, Korea) was used for kale cultivation. Kale seeds were sown in Rockwool cubes (W × L × H, 25 × 25 × 40 mm, Grodan Co., Roermond, Netherlands) and directly placed into the Tiiun. The light/dark cycle was 14:10 h, and light intensity during daytime (08:00–22:00) was set to level 5 out of 5 in the Tiiun system, and its actual light intensity was 8540 ± 320 lx. The temperature was adjusted to 26 °C and 18 °C for day and night, respectively. Thinning was performed in week 1, and kale was cultivated for five weeks and harvested. Pictures of the home vertical farming appliance (Tiiun) and a flow diagram of kale cultivation are presented in Fig. 1a and b.

### 2.2 Nutrient solution

To allow people to easily cultivate functional kale at home, a commercial nutrient solution product provided by a home vertical farming manufacturer (LG Electronics, Seoul, Korea) was adopted in the present study. The basic nutrient solution contained 53.61 ± 0.12 ppm P, 85.88 ± 0.26 ppm Ca, 19.97 ± 0.10 ppm Mg, 168.87 ± 0.52 ppm K, 33.38 ± 0.02 ppm S, and 138.5 ± 0.50 ppm N; and its electrical conductivity was 1.42 ms/cm and the pH was 6.4. The basic nutrient solution was initially supplied to all kale groups for three weeks, and the control (CON) group was provided with the basic nutrient solution two weeks later. Green tea water extract (GTE)-treated groups were provided with the basic nutrient solution for the initial three weeks, and then, the nutrient solution supplemented with 0.1 (0.1-GTE), 0.2 (0.2-GTE), 0.3 (0.3-GTE), or 0.5 (0.5-GTE) g/L of GTE was supplied for the last two weeks, respectively. To prepare GTE, 100 g of green tea powder (Danongwon, Eumseong, Korea) was mixed with 1 L of distilled water and extracted at 80 °C for 2 h, twice. The extract was then filtered using Whatman Grade 1 filter paper (Cytiva, Marlborough, MA, USA) and lyophilized. The yield of GTE was 20.98%.

### 2.3 Fresh and dry weights

A digital scale (HS224S; HANSUNG Instrument Co., Gwangmyeong, Korea) was used to measure the fresh and dry weights of kale plants. The fresh weight of kale was measured immediately after harvest, and its dry weight was measured after freeze-drying. The weight of kale was determined using the epigeal portion after removing the underground portion.

### 2.4 Mineral contents

The potassium and sodium contents of the kale samples were analyzed following the method described by Son et al. (2021) with minor adjustments. Lyophilized kale powder (0.05 g) was placed in a beaker, followed by the addition of 40 mL of 60% HNO_3_ solution. The solution was boiled for more than 2 h at 130 °C until it became transparent. The volume of the solution was adjusted to 50 mL with distilled water. The prepared sample solution was filtered using a 0.45-μm syringe filter, and its mineral contents were analyzed using an inductively coupled plasma optical emission spectrometer (ICP-OES) (OPTIMA 5300 DV; PerkinElmer, Waltham, MA, USA) at the BT Research Facility Center, Chung-Ang University.

### 2.5 Chlorophyll contents

The chlorophyll content of the kale samples was determined using the method described by Wellburn (1994) with minor modifications. One gram of freeze-dried kale powder was extracted with 5 mL of 80% acetone at 40 °C for 1 h, and the absorbance was measured at 663 and 646 nm using a spectrophotometer (SpectraMax M2e; Molecular Devices, San Jose, CA, USA). Chlorophyll content was determined using the following formula: chlorophyll *a* concentration = 12.25 × A_663_ − 2.79 × A_646_, chlorophyll *b* concentration = 21.5 × A_646_ − 5.1 × A_663_ (The “A” is for absorbance and subscripts denote wavelengths).

### 2.6 Color values

The color values of the kales were measured using a colorimeter (CM-36dG; KONICA MINOLTA, Tokyo, Japan). Freeze-dried kale powder (4 g) was placed in Petri dishes (35Φ), and the color was determined using the Hunter Lab color system. The light source was a D65 illuminant, with an observation angle of 10°.

### 2.7 Mechanical texture

A TA-XT ExpressC Texture Analyzer (Stable Micro Systems, Godalming, United Kingdom) was used to verify the mechanical texture of the fresh kale samples. Two types of texture analysis methods were used to analyze the textural characteristics. First, the parts of kale leaves were examined using a punching test with a P/20 cylinder probe. The compression mode was used with a pre-test speed of 5 mm/s, a test speed of 1 mm/s, and a post-test speed of 10 mm/s. The textural properties of the petioles were analyzed using a cutting test with an HDP/BS Knife-Edge probe. The compression mode was used for the analysis, and the pre-test, test, and post-test speeds were 5, 3, and 10 mm/s, respectively. The trigger force was set at 20 g for both tests.

### 2.8 Sensory evaluation

The sensory characteristics of kale were verified using quantitative descriptive analysis (QDA). Eight trained panelists developed three lexicons (bitterness, astringent, and grassy flavors) for kale samples and evaluated each characteristic using a 15 cm line scale. The panelists were recruited from Chung-Ang University: four were women, and four were men. Sensory evaluations were conducted in compliance with the ethical guidelines of the Institutional Review Board (IRB), and the entire study design was approved by the Chung-Ang University IRB Committee (1041078-202210-HR-246).

### 2.9 Antioxidative capacities

Freeze-dried kale powder (0.25 g) was mixed with 80% ethanol (5 mL) and extracted by sonication for 40 min at 40 °C (JAC-5020; Kodo Co., Hwaseong, Korea). The supernatant was collected after centrifugation, and the remaining residue was re-extracted twice, using 80% ethanol. The collected supernatants were filtered through a 0.2 μm syringe filter and then evaporated using a vacuum concentrator (SPD 2030; Thermo Fisher Scientific, Waltham, MA, USA). The DPPH radical scavenging activity was determined according to the method of Brand-Williams et al. (1995) and the ferric-reducing ability of plasma (FRAP) was examined based on the method of Benzie and Strain (1996). The DPPH and FRAP results for each sample were compared with those of the Trolox standard and are represented as the Trolox equivalent (TE). Total phenolic content (TPC) was measured using the Folin–Ciocalteu assay (Singleton and Rossi, 1965), and total flavonoid content (TFC) was measured according to the method described by Meda et al. (2005). The TPC and TFC in the kale samples were calculated using gallic acid equivalent (GAE) and catechin equivalent (CE), respectively, using each standard.

### 2.10 Contents of respective phenolic compounds

The respective phenolic compound contents in 80% ethanol kale extracts were determined using reverse-phase high-performance liquid chromatography (HPLC) (Agilent Infinity Series 1260; Agilent Technologies, Santa Clara, CA, USA) with a C18 ODS-AQ column (4.6 × 150 mm, 5 μm; YMC, Meridian, ID, USA) according to the modified method of Wang et al. (2013). The column temperature was 40 °C, and the mobile phase consisted of water with 0.1% formic acid (solvent A) and acetonitrile with 0.1% formic acid (solvent B). The ratios of solvents A to B were maintained as follows for gradient elution: 0 min, 95:5 (A: B); 10 min, 90:10; 35 min, 87:13; 45 min, 85:15; 50 min, 82:18; 60 min, 81:19; 70 min, 78:22; 75–76 min, 20:80; 80–85 min, 95:5. The flow rate was 1 mL/min and the injection volume was 10 μL. Absorbance spectra were recorded at 254 nm. Each peak was identified using the analytical standards for phenolic compounds.

### 2.11 Caffeine contents

The caffeine content of kale was determined using the method described by Sereshti and Samadi (2014) with slight modifications. Briefly, 0.25 g of freeze-dried kale powder was extracted twice with 5 mL of distilled water at 80 °C for 30 min and centrifuged at 2000 rpm for 10 min at 4 °C. The supernatant was collected and the total sample volume was adjusted to 10 mL using distilled water. The solvent was filtered using a 0.2 μm syringe filter, and caffeine contents in prepared extracts were analyzed using a reverse-phase HPLC (Agilent Technologies). The samples were separated using a C18 ODS-AQ column (YMC) at 25 °C. Water (solvent A) and acetonitrile (solvent B) were used as mobile phases, and the gradient was as follows: 0 min, 98:2; 5 min, 92:8; 10 min, 90:10; 25 min, 83:17; 33–36 min, 20:80; 40–45 min, 98:2 (A:B). The flow rate was 1 mL/min and the injection volume was 20 μL. Caffeine was detected at a wavelength of 280 nm.

### 2.12 Contents of glucosinolates

The glucosinolate content of the kale samples was analyzed following the modified method of Kim et al. (2018). Briefly, 0.1 g of freeze-dried kale powder was mixed with 2 mL of 70% methanol and boiled at 95 °C for 10 min. The solvents were immediately cooled on ice and centrifuged at 2000 rpm for 15 min at 4 °C. The supernatant was collected in a tube, and the residue was extracted again. Benzyl-GLS (1 mM, 0.1 mL) was added to the combined supernatant, and the total volume of the solution was adjusted to 4 mL with 70% methanol. To precipitate the proteins, 1.5 mL of the solution was transferred to a 2 mL microcentrifuge tube (MCT-200-C; Axygen, Union City, CA, USA), and 0.15 mL of a mixture of 1 M lead acetate and 1 M barium acetate (1:1) was added. The solution was centrifuged at 12,000 rpm for 5 min, and 1 mL of the supernatant was loaded onto a mini prep column filled with diethyl-aminoethyl (DEAE) Sephadex A-25 anion exchange resin that was pre-activated in 0.1 M sodium acetate buffer. After adding 0.2 mL of 0.1% purified arylsulfatase, the column was covered and incubated at room temperature for 18 h. Desulfo-glucosinolates were eluted twice with 0.5 mL of distilled water and filtered through a 0.2 μm syringe filter. The prepared samples were analyzed using reverse-phase HPLC (Agilent Technologies) with a C18 ODS-AQ column (YMC, USA). The mobile gradient condition was as follows: water, solvent A; acetonitrile, solvent B; 0 min, 99.5:0.5; 7 min, 98.5:1.5; 15 min, 90:10; 25 min, 80:20; 35–39 min, 70:30; 41–45 min, 99.5:0.5 (A:B). The flow rate was 1 mL/min, and the injection volume was 20 μL. The column temperature was 35 °C and the spectra of glucosinolates were recorded at 227 nm. Each peak was identified using an analytical glucosinolate standard. The glucosinolate content in the kale samples was quantified by comparing the peak areas with those of the internal standard benzyl-glucosinolate (Brown et al., 2002).

### 2.13 Statistical analysis

Statistical analyses were performed using IBM SPSS Statistics, version 28 (IBM, Armonk, NY, USA). One-way analysis of variance (ANOVA) and Duncan's multiple range test were used to compare the sample groups, and statistical differences within groups were determined at p < 0.05. The experimental results are presented as mean ± standard deviation (SD). Principal component analysis (PCA) and clustering analysis were conducted to visualize the heatmap using IBM SPSS Statistics version 28 (IBM, USA).

## 3 Results and discussion

### 3.1 Yields of GTE-treated kales and their K and Na contents

To verify the effect of green tea treatment on kale growth and yield, the fresh and dry weights of individual kale plants were measured (Fig. 1c and d). The height of kales in our study was similar to those reported by Metallo et al. (2018) (data not shown), and the fresh weight of kales ranged from 9.07 to 13.29 g/plant with no significant differences between the groups. This finding indicated that kales can be effectively cultivated in the home vertical farming appliance, and the treatment of green tea extract did not hinder the growth or decrease the yield of kales. Concerning dry weight, the CON group did not show significant differences compared with the treatment groups except for 0.2-GTE (p < 0.05), where 0.2-GTE showed higher dry weight than CON. These findings suggest that adding 0.1–0.5 mg/L of green tea extract in a nutrient solution does not alter kale growth rate and yield in the home vertical farming appliance.

K is an essential mineral that mediates photosynthesis, protein synthesis, and enzyme activation in the plant body (Idowu and Aduayi, 2006). Na is another essential mineral that maintains balance with potassium and regulates osmotic pressure in organisms (Nieves-Cordones et al., 2016). Therefore, the amounts of K and Na can regulate plant growth, and their amounts at harvest are important nutritional values in the human diet. K and Na contents in kale samples were 45.90–49.91 mg/g dry weight basis (DW) and 5.23–5.63 mg/g DW, respectively and none of them showed significant differences between the groups (p > 0.05; Fig. 1e and f). In a previous study, the K and Na contents of kale cultivated using conventional farming systems were approximately 30 and 2 mg/g DW, respectively (Ayaz et al., 2006; Kapusta-Duch et al., 2021), which were lower than those in this study. Hydroponic cultivation uses a nutrient solution that is fertile with minerals; therefore, using a nutrient solution can accumulate more minerals in plants (Lee et al., 2011). Although the K and Na contents in kales in this study were slightly higher than those in conventional kales, green tea treatment did not affect K and Na concentrations.

### 3.2 Chlorophyll contents and color values of GTE-treated kales

Chlorophyll is an important pigment that plays a role in photosynthesis and the production of primary carbon-based energy sources in plants. Chlorophyll content can indirectly estimate the nutritional status of plants (Gitelson et al., 2003). Table 1 presents the contents of chlorophyll *a* and *b* and their sum in GTE-treated kales. No significant differences in chlorophyll contents were observed between the sample groups (p > 0.05); however, the chlorophyll *a*, *b*, and total chlorophyll contents in this study were considerably lower than those for conventionally cultivated kales (0.86, 0.35, and 1.21 mg/g DW, respectively) (Korus, 2013). This may be caused by the adoption of home vertical farming appliances owing to their artificial light sources (Jung and Arar, 2023).

**Table 1:** Chlorophyll contents and Hunter Lab color values of green tea-treated kales.

Sample	Chlorophyll (mg/g dry basis)			Color values		
	Chlorophyll *a*	Chlorophyll *b*	Chlorophyll (a+b)	L (lightness)	a (redness)	b (yellowness)
CON	0.372 ± 0.014^NS^	0.271 ± 0.030^NS^	0.643 ± 0.044^NS^	47.03 ± 1.23^a^	−6.74 ± 0.33^b^	12.81 ± 0.59^b^
0.1-GTE	0.378 ± 0.012	0.271 ± 0.025	0.649 ± 0.037	48.28 ± 0.39^a^	−7.06 ± 0.16^c^	13.47 ± 0.39^a^
0.2-GTE	0.381 ± 0.014	0.285 ± 0.029	0.666 ± 0.042	45.56 ± 1.27^b^	−6.34 ± 0.15^a^	12.16 ± 0.58^c^
0.3-GTE	0.378 ± 0.008	0.269 ± 0.018	0.646 ± 0.026	47.43 ± 0.59^a^	−6.90 ± 0.13^bc^	13.09 ± 0.35^ab^
0.5-GTE	0.377 ± 0.004	0.275 ± 0.009	0.659 ± 0.011	47.11 ± 1.27^a^	−6.69 ± 0.24^b^	12.98 ± 0.42^ab^

Data represent the means ± SDs (n = 6, biologically independent plant sample groups).

Different letters in the same column indicate significant differences (p < 0.05). NS, not significant.

For color values, 0.1-GTE exhibited the highest L (brightness) and b (yellowness) values and the lowest a (redness) value (Table 1), and 0.2-GTE showed the opposite trends with 0.1-GTE. However, no consistent patterns were observed across the GTE treatments.

### 3.3 Texture properties and sensory characteristics of GTE-treated kales

Table 2 lists the mechanical texture characteristics of two kale parts (leaf and petioles). The punching (leaf) and cutting (petiole) test methods were applied because of the different textural properties of the leaf and petiole. Consequently, the hardness of kale leaf ranged from 50.03 to 59.98 g, and no significant differences were observed between the groups. In the case of the petiole part, samples from the 0.2-GTE group had the hardest texture with 503.62 ± 156.90 g, but we could not find an apparent tendency between hardness and the concentration of green tea extract.

**Table 2:** Mechanical texture properties and sensory characteristics of green tea-treated kales.

Sample	Texture (g)		Sensory characteristics		
	Cutting	Punching	Bitterness	Astringent	Grassy flavor
CON	319.37 ± 96.46^b^	59.98 ± 12.45^NS^	8.65 ± 3.12^NS^	5.63 ± 2.68^ab^	8.74 ± 1.44^NS^
0.1-GTE	384.07 ± 66.38^ab^	52.96 ± 8.83	7.66 ± 3.66	4.60 ± 2.45^b^	7.96 ± 2.83
0.2-GTE	503.62 ± 156.90^a^	50.03 ± 5.78	8.46 ± 2.53	8.56 ± 2.67^a^	8.08 ± 3.76
0.3-GTE	366.63 ± 53.34^b^	52.86 ± 6.05	7.80 ± 2.75	7.63 ± 2.20^ab^	6.94 ± 2.93
0.5-GTE	320.52 ± 79.44^b^	52.36 ± 4.01	8.11 ± 3.54	5.51 ± 3.01^ab^	6.65 ± 2.86

Mechanical texture properties were examined with six biologically independent plant samples, and eight trained panelists determined sensory characteristics.

Data represent the means ± SDs; different letters in the same column indicate significant differences (p < 0.05). NS, not significant.

To evaluate the sensory attributes of GTE-treated kales, bitterness, astringency, and grassy flavor were assessed using QDA (Table 2). No significant differences were found in bitterness and grassy flavor between CON and GTE-treated groups. These findings suggest that treatment with green tea extract during kale cultivation did not negatively affect kale qualities, such as yield, mineral content, chlorophyll content, color, texture, and sensory traits.

### 3.4 Antioxidative capacities of GTE-treated kales

**Fig. 2:**
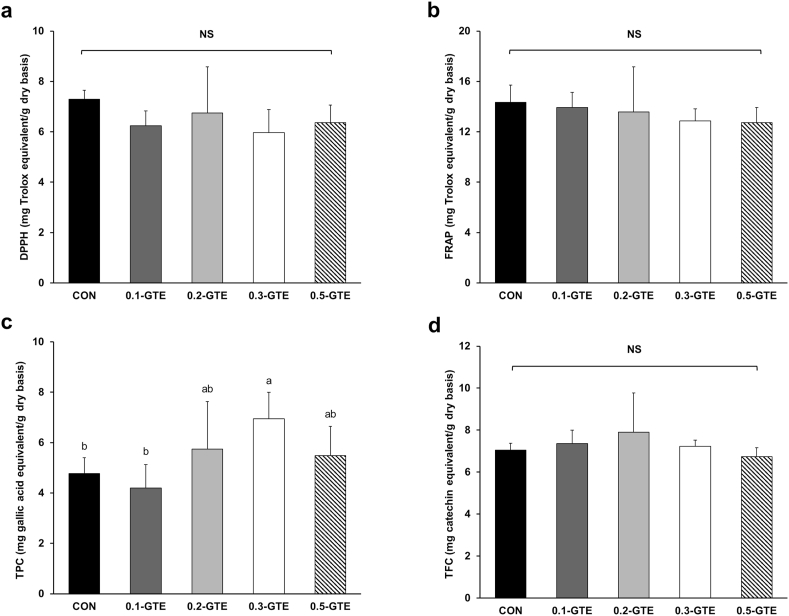
Antioxidant capacities of green tea-treated kales. DPPH (a), FRAP (b), total phenolic content (c), and total flavonoid content (d) were examined. Data represent the means ± SDs (n = 6, biologically independent plant sample groups). Different letters indicate significant differences within groups (p < 0.05). NS, not significant.

The antioxidative capacity of GTE-treated kales was assessed using DPPH, FRAP, TPC, and TFC analyses (Fig. 2). GTE-treated kales showed 5.96–7.30 and 12.72–14.35 mg TE/g DW for DPPH and FRAP, and no significant differences within samples were observed in both experiments. DPPH radical scavenging activity was lower than kales cultivated by conventional farming, 9.61 mg TE/g DW (Lotti et al., 2018) and 11.11 mg TE/g DW (Ilyasoğlu and Burnaz, 2015), and this trend was also detected in FRAP activity (Leamsamrong et al., 2019). The decreased antioxidative capacities of kales cultivated using vertical farming systems may be due to the intensity of exposure to extrinsic stress factors. The defensive response in plants primarily mediates the production of secondary metabolites related to antioxidative activities (Scheible et al., 2004). Vertical farming systems precisely adjust environments with luxuriant nutrient supplies and effectively obstruct the invasion of insects and microorganisms (Avgoustaki and Xydis, 2020; Oh and Lu, 2023). These conditions can ease the stress on plants in vertical farming, which may have resulted in the lowered antioxidative capacities of kale cultivated on vertical farms.

Concordant with the results of DPPH and FRAP assays, GTE treatment did not affect TFC in kale. However, the TPC of samples from the 0.3-GTE group was significantly higher than that of the samples from the CON and 0.1-GTE groups (p < 0.05). These findings are consistent with those of a study by Ertani et al. (2011), who demonstrated that the addition of materials with high TPCs, such as blueberry and apple hydrolysates, to nutrient solutions could increase the TPC in corn leaves. However, in our study, treatment with GTE did not notably alter the antioxidative capacities of kales. This finding contradicted our hypothesis that green tea treatment would enhance the antioxidative properties of kale owing to its high TPC and TFC.

### 3.5 Changes in phenolic components and caffeine amounts of GTE treatment of kale

**Fig. 3:**
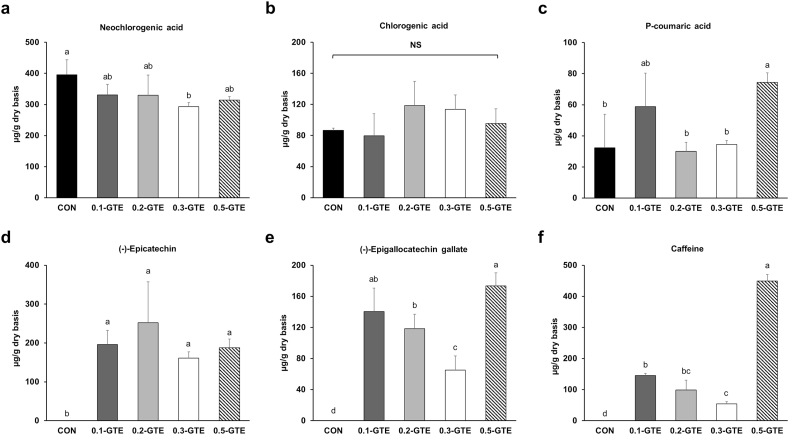
Contents of respective phenolic compounds in green tea-treated kales and the caffeine amount. Neochlorogenic acid (a), chlorogenic acid (b), p-coumaric acid (c), epicatechin (d), epigallocatechin gallate (e), and caffeine (f) were quantified with HPLC. Data represent the means ± SDs (n = 3, biologically independent plant sample groups). Different letters indicate significant differences within groups (p < 0.05). NS, not significant.

First, we used HPLC to analyze the phenolic and caffeine family compounds in GTE. GTE was rich in gallic acid and various catechins, such as (+)-catechin, (−)-epigallocatechin (EGC), (−)-epicatechin (EC), (−)-epigallocatechin gallate (EGCG), and (−)-epicatechin gallate (ECG) (Supplementary Table 1). In contrast, the primary phenolic acids in kale were neochlorogenic, chlorogenic, and p-coumaric acids, with catechins naturally absent in untreated kale (Fig. 3). Neochlorogenic acid was the most abundant phenolic compound in CON (395.55 ± 47.96 μg/g DW), followed by chlorogenic acid (86.79 ± 2.60 μg/g DW) and p-coumaric acid (32.35 ± 21.45 μg/g DW). GTE decreased neochlorogenic acid, with 0.3-GTE showing significantly lower neochlorogenic acid content (292.74 ± 13.80 μg/g DW) than CON (p < 0.05). However, the chlorogenic acid content remained unaffected, while p-coumaric acid content was significantly elevated in the 0.5-GTE group compared to that in the CON group (p < 0.05). Moreover, treatment with green tea introduced catechins (EC and EGCG) into kale. In the treated groups, EC levels ranged from 160.81 to 252.11 μg/g DW, and those of EGCG ranged from 64.97 to 173.26 μg/g DW. These findings demonstrate that EC and EGCG were absorbed and accumulated in kale as a result of green tea treatment during kale cultivation. Phenolic compounds are secondary plant metabolites that are abundant in plant-based foods and have diverse bioactivities (Meinhart et al., 2019). Epidemiological studies suggest that prolonged consumption of phenol-rich foods can impede the occurrence of diabetes, cancer, and cardiovascular diseases (Nardini, 2022; Sun and Shahrajabian, 2023). Green tea treatment elevated the TPC and introduced the additional bioactive compounds, EC and EGCG, in kale. Catechin-rich kale may exhibit enhanced health benefits, as catechins are known for their protective effects against inflammatory disorders, glucose and lipid metabolic disorders, coronary diseases, and degenerative diseases (Chacko et al., 2010; Crespy and Williamson, 2004).

Green tea extracts generally contain five major catechin compounds: (+)-catechin, (−)-EC, (−)-EGC, (−)-ECG, and (−)-EGCG (Grzesik et al., 2018), consistent with the green tea extract used in this study. However, only two types of catechins (EC and EGCG) were detected in green tea-treated kale. Despite the known health benefits of catechins, their absorption and bioavailability in the human body are generally low. Therefore, strategies such as nanostructure-based carriers and molecular modifications of catechins have been explored to enhance catechin uptake (Cai et al., 2018). Although studies on catechin absorption mechanisms in plant roots are lacking, our findings, along with those of a study by Cai et al. (2018) indicate that the absorption efficiency of each catechin may vary. Sirk et al. (2008) observed distinct molecular dynamics between EC and EGCG and other catechins, such as EGC and ECG, during their interactions with cellular lipid bilayers. Compared with EGC and ECG, which predominantly adhered to the bilayer surface, EC and EGCG penetrated the surface and were located beneath the bilayer, possibly due to higher affinities with lipid headgroups by multiple hydrogen bonds. This difference in membrane penetration may explain the efficient absorption and accumulation of EC and EGCG in kale plants. The abundance of hydrogen bonds is also related to the biological activities of catechins; EGCG forms numerous hydrogen bonds due to its gallate moiety (Sirk et al., 2009). This underlies the superior bioactivity of EGCG among catechins (Coimbra et al., 2006), and the abundant EGCG content in GTE-treated kales could result in strong potency. Further studies are required to understand the mechanisms of absorption, distribution, metabolism, and excretion of green tea catechins from kale bodies.

Caffeine is a naturally occurring alkaloid found in various plants, particularly coffee and green tea. An intake of caffeine improves exercise performance and promotes fat breakdown; however, the excess intake causes some side effects like anxiety, digestive trouble, and sleep disturbances owing to its stimulating effect (Saraiva et al., 2023). The green tea extract prepared in this study contained 3.28 mg/g DW of caffeine, but it did not entirely accumulate in kale. Caffeine contents in GTE-treated kales ranged from 54.40 to 449.49 μg/g DW (Figs. 3f), and 0.5-GTE contained significantly higher caffeine amounts than other treatment groups (p < 0.05). The caffeine content in GTE-treated kales was much lower than the maximum safe daily dosage of caffeine for adults released by the European Food Safety Authority (EFSA), which is 400 mg (equivalent to 5.7 mg/kg body weight for a 70 kg adult). In other words, approximately 1 kg of dried 0.5-GTE is required to exceed the daily caffeine criteria. Therefore, the risk of side effects due to caffeine in GTE-treated kale was negligible.

### 3.6 Glucosinolate contents in GTE-treated kales

**Fig. 4:**
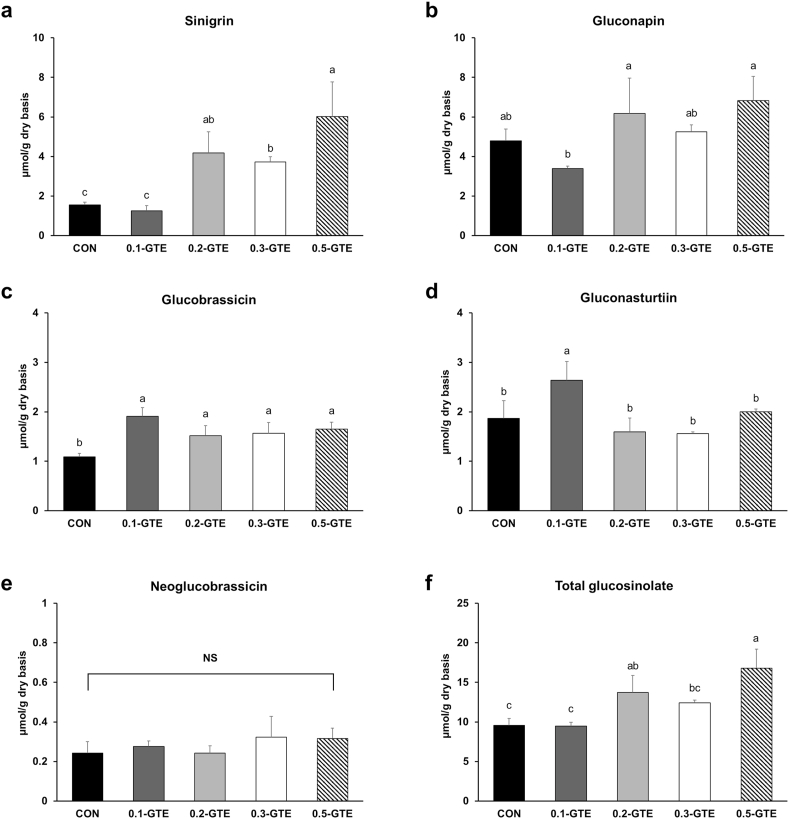
Glucosinolate contents in green tea-treated kales. The contents of sinigrin (a), gluconapin (b), glucobrassicin (c), gluconasturtiin (d), neoglucobrassicin (e), and total glucosinolate (f) were determined. Data represent the means ± SDs (n = 3, biologically independent plant sample groups). Different letters indicate significant differences within groups (p < 0.05). NS, not significant.

Glucosinolates are sulfur-rich anionic secondary metabolites primarily found in cruciferous plants, including kale (Clarke, 2010). Upon ingestion, glucosinolates are hydrolyzed to various bioactive products, such as isothiocyanates and indole-3-carbinols, which can stimulate the activities of phase I and II detoxification enzymes in the human body (Cartea and Velasco, 2008; Hayes et al., 2008). Glucosinolates also exhibit anti-inflammatory and antioxidant activities and are potent anticancer agents (Cartea et al., 2008; Connolly et al., 2021). Glucosinolates are divided into three main categories, depending on their precursor compounds: aliphatic (methionine), indole (tryptophan), and aromatic (phenylalanine) (Katsarou et al., 2016). In the present study, five glucosinolates (sinigrin, gluconapin, glucobrassicin, gluconasturtiin, and neoglucobrassicin) were identified in the GTE-treated kale samples (Fig. 4). Among these, two (sinigrin and gluconapin) were aliphatic, two (glucobrassicin and neoglucobrassicin) were indole, and one (neoglucobrassicin) was an aromatic glucosinolate. The most abundant glucosinolate compound in CON kale was gluconapin (4.80 ± 0.58 μmol/g DW), followed by sinigrin, gluconasturtiin, glucobrassicin, and neoglucobrassicin. The GTE treatment significantly increased sinigrin content, which was approximately four times higher in the 0.5-GTE group than in the CON group. The content of glucobrassicin was also significantly increased in the GTE-treated groups (p < 0.05). Consequently, the total glucosinolate content in the 0.5-GTE group reached 16.81 ± 2.38 μmol/g DW, which was almost two-fold higher than that in the CON (9.56 ± 0.90 μmol/g DW; p < 0.05). These findings suggest that green tea treatment not only enriches the kale samples with catechins but also enhances the content of glucosinolates, which are inherent bioactive compounds in kale. Collectively, the combined bioactivities of catechins and augmented glucosinolates are anticipated to provide greater health benefits when consuming GTE-treated kales.

Similar to other secondary metabolites, the rate of glucosinolate biosynthesis in plants is an integrated output of the biotic and abiotic conditions during cultivation (Grubb and Abel, 2006). Therefore, the controlled environment of vertical farming enables us to obtain more optimized secondary metabolite content from plants (Bafort and Jijakli, 2024). In addition, vertical farming systems can easily accommodate the application of elicitors that regulate the biosynthesis of secondary metabolites. Because secondary metabolism in plants is part of the defense system against stress factors, elicitors have been shown to stimulate the production of secondary metabolites (Aguirre-Becerra et al., 2021). In this study, the application of green tea extract as an elicitor in home vertical farming appliances successfully increased glucosinolate synthesis in kale, particularly, aliphatic glucosinolates. Aliphatic glucosinolates are derived from methionine and undergo molecular changes in core structure and side chain via a synthetic pathway involving more than 20 genes (Mitreiter and Gigolashvili, 2021). The transcription factors *MYB28* and *MYB29* are specifically associated with the biosynthesis of aliphatic glucosinolates and regulate the expression of several genes involved in converting methionine to desulfoglucosinolate (Yi et al., 2015). Therefore, green tea treatment is anticipated to promote the expression of a series of metabolic pathways related to aliphatic glucosinolate biosynthesis; however, further studies are required to investigate the changes in the expression of the genes related to this process.

### 3.7 Heatmap analysis and PCA results of GTE-treated kales

**Fig. 5:**
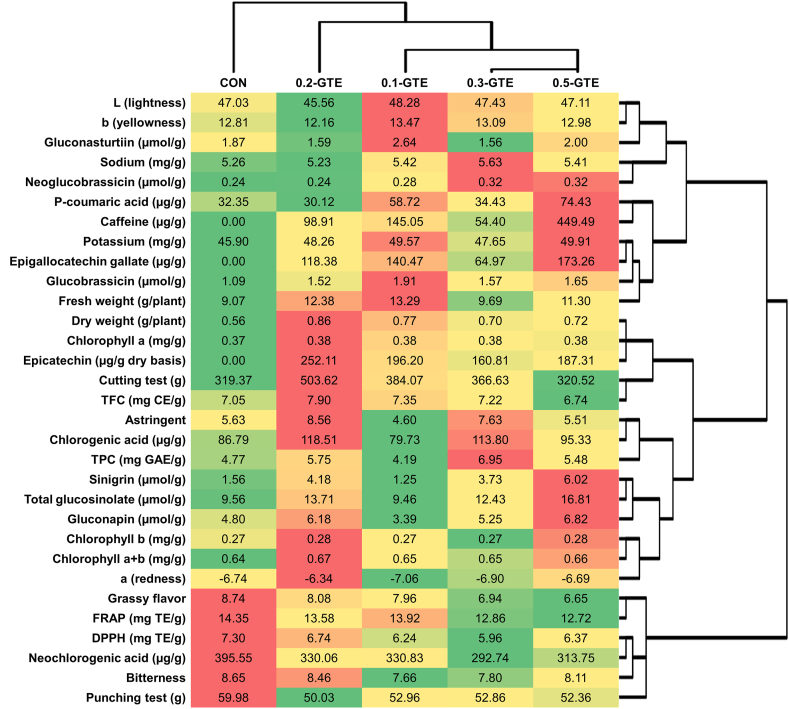
Heatmap analysis of variables of green tea-treated kales. The magnitudes of values are represented using green (minimum) to red (maximum) colors. (For interpretation of the references to color in this figure legend, the reader is referred to the Web version of this article.)

**Fig. 6:**
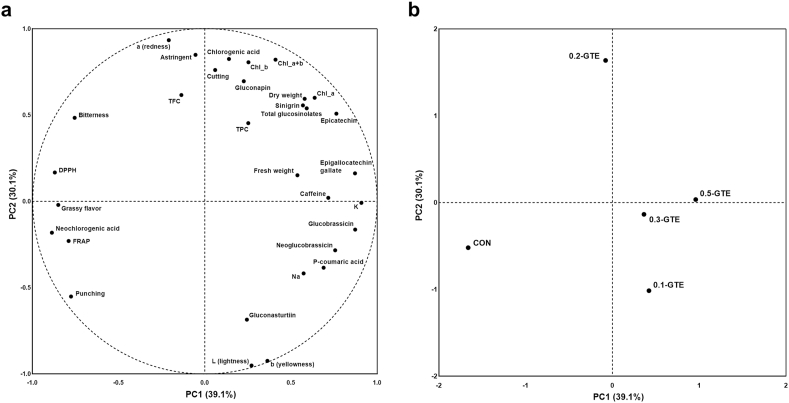
Principal component analysis (PCA) plot of green tea-treated kales. PCA plots were visualized using variables (a) and sample groups (b).

Heatmap analysis and PCA were conducted to analyze and visualize the relationship between the variables and sample groups (Fig. 5, Fig. 6). In the PCA results, PC1 and PC2 presented low explained variance ratios (39.1% and 30.1%, respectively), which may be because variables unchanged by green tea treatment were primarily associated with food quality factors such as yield, color, texture, and sensory traits. Despite the limited explained variance ratio, differences in the overall characteristics between the CON and 0.5-GTE groups were prominent in the PCA plot and heatmap. Among the treatment groups, samples from the 0.3-GTE and 0.5-GTE groups showed the most similar characteristics and were distinguishable from those in the CON group in terms of EC, EGCG, caffeine, sinigrin, and total glucosinolate contents. These findings suggest that GTE treatment during kale cultivation effectively fortified certain secondary metabolites and may be suitable for producing tailored kale with improved health benefits.

## 4 Conclusion

This study aimed to investigate the production of kales with enhanced bioactivity using home vertical farming appliances. GTE treatment (up to 0.5 g/L) during kale cultivation did not impede its yield (fresh and dry weight) nor did it significantly alter the Na and K contents, color, texture, and sensory characteristics were also not notably changed by green tea treatment. In comparison, the green tea treatment slightly enhanced the TPC; in particular, some alien chemical compounds were absorbed from green tea to kale. Although catechins were not initially present in kale, after green tea treatment, two types of catechins (EC and EGCG) were found to have been efficiently absorbed into kale. Green tea treatment supplemented extrinsic chemical compounds in kales and enhanced the biosynthesis of glucosinolate, a unique secondary metabolite of kales, increasing the total glucosinolate content in kale by two-fold; this may enhance the anticancer effects of kale. Caffeine was also infused into kale during cultivation; however, its content was marginal to cause side effects in the normal diet. In summary, the GTE treatment fortified kale with bioactive compounds without compromising its quality. These findings highlight the potential of adjusting specific nutrients or functional substances to obtain personally tailored harvests. Furthermore, because a home vertical farming appliance was tested as a cultivator in the present study, we anticipate that our results will enable people to grow personally tailored plants with strong bioactivity in their homes.

## CRediT authorship contribution statement

**Young-Woong Ju:** Investigation, Validation, Formal analysis, Methodology, Data curation, Writing – review & editing, Writing – original draft. **Su-Hyeon Pyo:** Investigation, Validation, Formal analysis, Methodology, Data curation. **So-Won Park:** Investigation, Validation, Formal analysis, Methodology, Data curation. **Chae-Ryun Moon:** Investigation, Validation, Formal analysis, Methodology, Data curation. **Seul Lee:** Investigation, Validation, Formal analysis, Methodology, Data curation. **Mzia Benashvili:** Investigation, Validation, Formal analysis, Methodology, Data curation. **Jai-Eok Park:** Investigation, Conceptualization, Data curation. **Chu Won Nho:** Investigation, Conceptualization, Data curation. **Yang-Ju Son:** Project administration, Investigation, Validation, Conceptualization, Formal analysis, Methodology, Data curation, Writing – review & editing, Writing – original draft, Funding acquisition, Supervision.

## Declaration of competing interest

The authors declare that they have no known competing financial interests or personal relationships that could have appeared to influence the work reported in this paper.

## Data Availability

Data will be made available on request.
