# Supplementary material for: Treatment of water extract of green tea during kale cultivation using a home vertical farming appliance conveyed catechins into kale and elevated glucosinolate contents
Source: Curr Res Food Sci. 2024 Sep 14;9:100852. doi: 10.1016/j.crfs.2024.100852 (PMC11421350; doi:10.1016/j.crfs.2024.100852)
Supplement: Multimedia component 1 [file mmc1.docx]

**Supplementary Table 1.** Content of phenolic compounds and caffeine in the water extracts of green tea.

| **Compounds** | **Contents (mg/g dry basis)** |
| --- | --- |
| **Gallic acid** | 0.06 |
| **(-)-Epigallocatechin** | 2.69 |
| **(+)-Catechin** | 0.56 |
| **(-)-Epicatechin** | 0.68 |
| **(-)-Epigallocatechin gallate** | 0.86 |
| **(-)-Epicatechin gallate** | 0.56 |
| **Caffeine** | 3.28 |
